# Sestrin2 regulates microglia polarization through mTOR-mediated autophagic flux to attenuate inflammation during experimental brain ischemia

**DOI:** 10.1186/s12974-020-01987-y

**Published:** 2020-11-05

**Authors:** Tingting He, Wanlu Li, Yaying Song, Zongwei Li, Yaohui Tang, Zhijun Zhang, Guo-Yuan Yang

**Affiliations:** 1 Department of Neurology, Ruijin Hospital, School of Medicine, Shanghai Jiao Tong University, Shanghai, 200025 China; 2 School of Biomedical Engineering and Med-X Research Institute, Shanghai Jiao Tong University, Shanghai, 200000 China

**Keywords:** Autophagic flux, Inflammation, Ischemia, Microglia, mTOR

## Abstract

**Background:**

Neuroinflammation is the major pathogenesis of cerebral ischemia. Microglia are activated and polarized to either the pro-inflammatory M1 phenotype or anti-inflammatory M2 phenotype, which act as a critical mediator of neuroinflammation. Sestrin2 has pro-survival properties against ischemic brain injury. However, whether sestrin2 has an anti-inflammatory function by shifting microglia polarization and its underlying mechanism is unknown.

**Methods:**

Adult male C57BL/6 mice (*N* = 108) underwent transient middle cerebral artery occlusion (tMCAO) and were treated with exogenous sestrin2. Neurological deficit scores and infarct volume were determined. Cell apoptosis was examined by TUNEL staining and Western blotting. The expression of inflammatory mediators, M1/M2-specific markers, and signaling pathways were detected by reverse transcription-polymerase chain reaction, immunostaining, and Western blotting. To explore the underlying mechanism, primary neurons were subjected to oxygen-glucose deprivation (OGD) and then treated with oxygenated condition medium of BV2 cells incubated with different doses of sestrin2.

**Results:**

Sestrin2 attenuated the neurological deficits, infarction volume, and cell apoptosis after tMCAO compared to those in the control (*p* < 0.05). Sestrin2 had an anti-inflammatory effect and could suppress M1 microglia polarization and promote M2 microglia polarization*.* Condition medium from BV2 cells cultured with sestrin2 reduced neuronal apoptosis after OGD in vitro. Furthermore, we demonstrated that sestrin2 drives microglia to the M2 phenotype by inhibiting the mammalian target of rapamycin (mTOR) signaling pathway and restoring autophagic flux.

**Conclusions:**

Sestrin2 exhibited neuroprotection by shifting microglia polarization from the M1 to M2 phenotype in ischemic mouse brain, which may be due to suppression of the mTOR signaling pathway and the restoration of autophagic flux.

## Background

Ischemic stroke is characterized by a high rate of disability and death, and is largely prevalent in China [1, 2]. Despite the narrow therapeutic window, thrombolysis and endovascular therapy could effectively improve patients’ clinical outcomes. However, reperfusion injury after recanalization deteriorates outcomes due to lesion growth and hemorrhagic transformation [3, 4]. New therapeutic strategies are needed to alleviate ischemic brain injury during the acute phase of stroke.

Post-ischemic inflammation, elicited by dying cells and debris due to an initial ischemic insult, is the key mechanism behind secondary degeneration in an ischemic brain [5]. Microglia, the major resident immune cells in the central nervous system, are the first cells that respond to ischemic insult. Activated microglia are important for post-ischemic inflammation, which has long been considered a negative factor contributing to stroke outcomes [6]. Microglia play a dual role in brain injury, repair, and regeneration after ischemic stroke [7]. They could polarize into two distinct phenotypes, the “classically activated” M1 phenotype and the “alternatively activated” M2 phenotype, which regulate the post-ischemic micro-environment [8, 9]. M1 microglia are pro-inflammatory and secrete pro-inflammatory mediators such as tumor necrosis factor-α (TNF-α), interlukin-1β (IL-1β), IL-6, and interferon-γ (IFN-γ). They also secrete reactive oxygen/nitrogen species and proteolytic enzymes such as matrix metalloproteinase-9, which exacerbate inflammation and lead to tissue damage. In contrast, M2 microglia secrete anti-inflammatory mediators such as IL-10, transforming growth factor-β (TGF-β), IL-4, IL-13, and insulin-like growth factor-1 (IGF-1). They can also secrete growth factors such as vascular endothelial growth factor, brain-derived neurotrophic factor, and clear cell debris, which suppress excessive inflammation and promote tissue recovery [10]. The balance between the M1 and M2 states is dynamic during the progression of ischemic stroke. M2 microglia predominate at the early stage of disease but are dampened during the repair process, while M1 constantly accumulates [10, 11]. However, the switch from the M2 to M1 phenotype may create an unfavorable microenvironment. Regulation of the balance between M1 and M2 phenotypes is a promising therapeutic strategy for ischemic stroke.

Sestrin2, a stress sensor protein, can respond to various injuries such as energy deficiency, oxidative stress, DNA damage, and hypoxia, to maintain metabolic homeostasis [12]. Increasing evidence has shown that sestrin2 has pro-survival properties after ischemic injury [13]. Recent studies have revealed that sestrin2 acts as a scaffold protein that mediates adenosine 5′-monophosphate-activated protein kinase (AMPK) activation to protect against myocardial ischemia through interacting with the upstream factor liver kinase B1 (LKB1) [14]. Intra-nasal administration of recombinant human sestrin2 (rh-sestrin2) protects from neonatal hypoxic ischemic encephalopathy in rats, by modulating the AMPK/mammalian target of rapamycin (mTOR) pathway [15]. Sestrin2 over-expression has been shown to increase NF-E2-related factor 2 (Nrf2)/heme oxygenase-1 (HO-1) pathway-mediated angiogenesis following focal brain ischemia [16]. However, it remains unclear how sestrin2 modulates post-ischemic inflammation. Sestrin2 has also been shown to inhibit Toll-like receptor-induced pro-inflammatory signaling and protect RAW264.7 (macrophage cell line) cells from apoptosis by inhibiting mitogen-activated protein kinase (MAPK) phosphorylation [17]. Furthermore, Sestrin2 has been shown to suppress sepsis in macrophages by inducing mitophagy and inhibiting NLRP3 activation [18]. Sestrin2 has been shown to regulate the cardiac macrophage-mediated inflammatory response after myocardial infarction by inhibiting the pro-inflammatory response of M1 macrophages [19]. As “resident macrophages” in the brain, microglia are part of the mononuclear phagocyte system. However, microglia and macrophages have distinct cellular origins and unique temporal and spatial presence in the context of stroke, suggesting distinctive and complementary roles [20]. Whether sestrin2 has a phenotype switching effect on microglia is unknown.

To answer these questions, we planned to explore (1) whether sestrin2 modulates the microglial phenotype switch to suppress inflammation in the acute phase of stroke, and (2) what the underlying mechanism of sestrin2 effects is. Our results may provide useful information for the development of a novel immunomodulatory therapeutic approach for acute ischemic stroke.

## Methods

### Cell culture

The immortalized BV2 microglial cell line was cultured in complete Dulbecco’s modified Eagle medium (DMEM, HyClone, Logan, UT, USA) supplemented with fetal bovine serum (10%; Gibco, Carlsbad, NM, USA) and penicillin/streptomycin antibiotics (1%; HyClone).

Primary neuronal cultures were prepared from 16-day-old embryos of ICR mice (Jiesijie, Shanghai, China). Briefly, the cortex was isolated and dissociated by trypsin (HyClone). The pellets were filtered through 70 μm strainers (Millipore, Billerica, MA, USA) and suspended after centrifugation. Following this, cells were seeded on dishes coated with poly-d-lysine (Sigma-Aldrich, St. Louis, MO, USA) and cultured in DMEM for 4 h. The medium was then changed to Neurobasal (Gibco) medium containing B-27 (2%; Gibco). Cell culture medium was changed every 3 days and used for the following experiments 6 to 10 days after seeding.

### Oxygen-glucose deprivation and re-oxygenation model and drug treatment

A sealed anaerobic chamber infused with a gas mixture of 95% N_2_ and 5% CO_2_ was used for oxygen-glucose deprivation (OGD). BV2 cell culture medium was replaced with deoxygenated glucose-free DMEM (Gibco) with or without rh-sestrin2 (6.25–100 ng/ml; Sigma-Aldrich) and MHY-1485 (5 μM; Selleck, Shanghai, China, S7811). Then, BV2 cells were subjected to OGD for 3 h in the chamber, then the cultures was replaced by DMEM and underwent re-oxygenation for 12 h.

Primary neurons were subjected to OGD for 45 min and then treated with their maintenance medium or 50% BV2 re-oxygenation condition medium (BV2-CM). Cells were then allowed to recover for 24 h during re-oxygenation.

### Lactate dehydrogenase release assay

Viability of primary neurons after OGD was assessed using an lactate dehydrogenase (LDH) assay kit (Beyotime, Shanghai, China) following the manufacturer’s protocol. LDH levels were measured in the supernatants of neurons after re-oxygenation. Absorbance was measured at 490 nm using a microplate reader (BioTek, Winooski, VT, USA).

### Transient middle cerebral artery occlusion surgery

All animal procedures were approved by the Institutional Animal Care and Use Committee of Shanghai Jiao Tong University, Shanghai, China. Reporting of these experiments complied with the ARRIVE (Animal Research: Reporting in Vivo Experiments) guidelines.

**Fig. 1:**
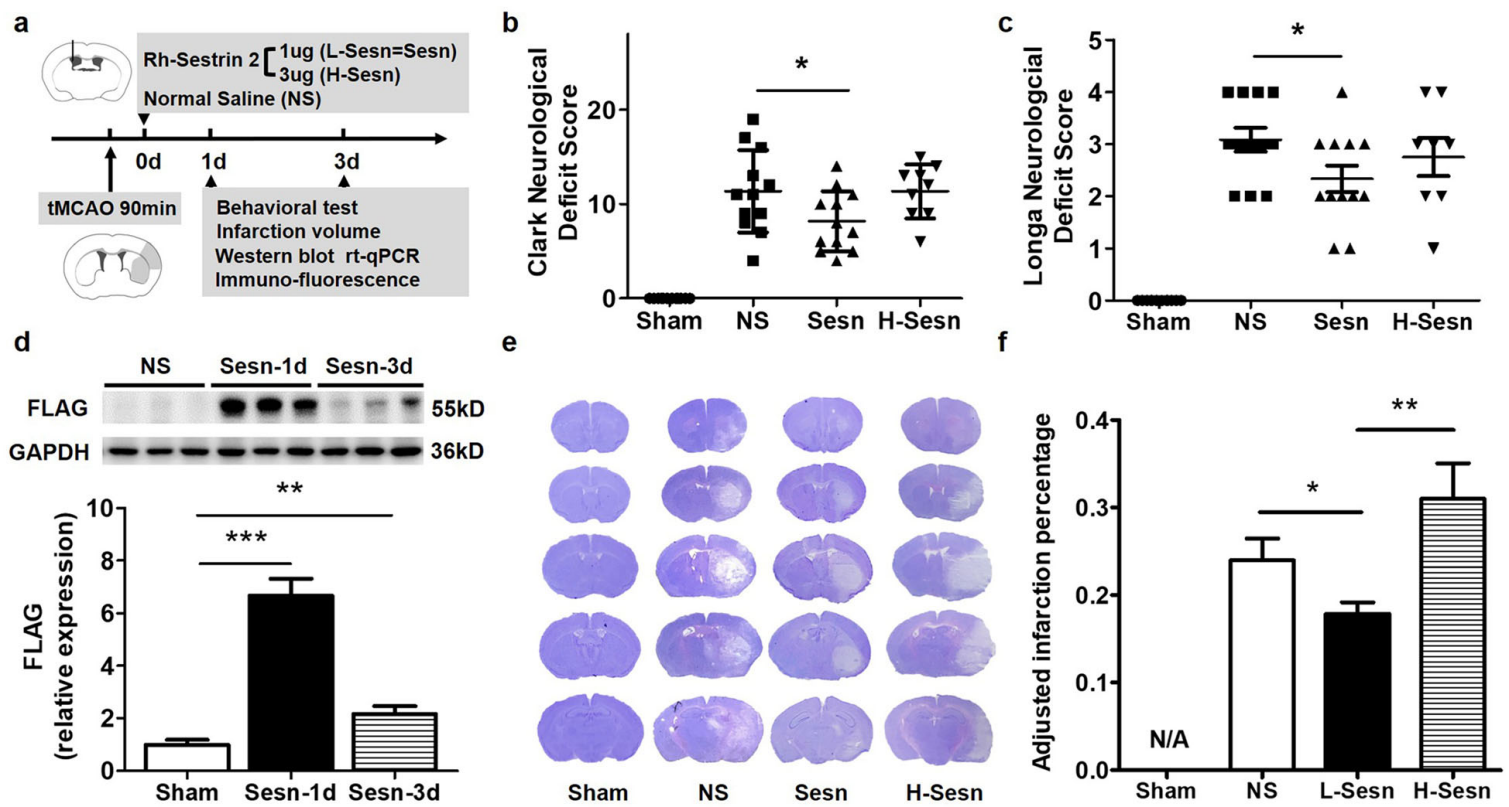
Sestrin2 improved neurological functional recovery after tMCAO. **a** The timeline of the experimental protocol. **b**, **c** Bar graphs show the results of Clark and Longa neurological deficit scores at 1 day after tMCAO in Sham, NS, Sesn, and H-sesn groups. *N* = 10 per group. Data are presented as mean ± SEM, *p < 0.01, NS vs. Sesn. **d** Western blot and the quantification of FLAG-tagged sestrin2 expression in the Sesn-1dai and Sesn-3day groups. *N* = 3 per group. Data are presented as mean ± SEM, ***p < 0.001, NS vs. Sesn-1d, **p < 0.01, NS vs. Sesn-3d. **e** Representative images for cresyl violet staining of brain slices at 1day after tMCAO. **f** Quantification of adjusted infarction percentage based on the results of cresyl violet staining. N/A = not applicable. *N* = 10 per group. Data are presented as mean ± SEM, *p < 0.05, NS vs. Sesn, **p < 0.01, Sesn vs. H-Sesn

The experimental design is shown in Fig. 1a. A total of 108 8-week-old male C57BL/6 mice (25–30 g; Jiesijie) were used for the experiments. Animals were randomly divided into 4 groups: sham group, normal saline-treated group (NS), 1 mg rh-sestrin2-treated group (Sesn), and 3 μg rh-sestrin2-treated group (H-Sesn). After transient middle cerebral artery occlusion (tMCAO), NS, Sesn, and H-Sensn mice underwent stereotactic injection of normal saline, 1 μg rh-sestrin2, or 3 μg rh-sestrin2 into the lateral ventricle, respectively. The tMCAO surgery was then performed as previously described [21]. In brief, mice were anesthetized with 1.5% isoflurane (RWD Life Science, Shenzhen, China) in a 30/68.5% oxygen/nitrous oxide mixture. A 6-0 suture (Covidien, Mansfield, MA, USA) coated with silicon was inserted into the middle cerebral artery through an external carotid artery incision. Cerebral blood flow was monitored using laser Doppler flowmetry (Moor Instruments, Devon, UK), and successful occlusion was marked by more than 80% decline. After 90 min of occlusion, the suture was withdrawn to allow reperfusion. Sham-operated mice underwent the same procedure except for filament insertion. Animals were sacrificed at 1 and 3 days after surgery.

### Neurobehavioral tests

Functional deficiency was assessed at 1 and 3 days after surgery and conducted by an investigator blinded to the experimental design. The Clark neurological deficit score (0–28) and Longa neurological deficit score (0–5), which have positive correlations with functional deficits, were calculated as previously described [22, 23].

### Infarct volume assessment

Mouse brains were firstly perfused with 0.9% saline and then frozen rapidly in − 40 °C isopentane (Sinopharm Chemical Reagent, Shanghai, China). Twenty-micrometer-thick frozen brain sections from the anterior commissure to the hippocampus were obtained by a cryostat microtome (Leica, Wetzlar, Germany). The infarct volume was determined by Cresyl Violet acetate (0.05%; Sigma-Aldrich) staining and calculated as an adjusted percentage to compensate for the effect of brain edema using the following formula: {[total infarct volume − (the volume of ipsilateral hemisphere − the volume of contralateral hemisphere)] / contralateral hemisphere volume} × 100% [24].

### Immunostaining

Mouse brains were obtained under deep anesthesia 1 and 3 days after surgery. They were first perfused with 0.9% saline and then fixed with 4% paraformaldehyde (PFA, Sinopharm Chemical Reagent, Shanghai, China). After being fully dehydrated in 30% sucrose, 30-μm-thick brain sections from the anterior commissure to the hippocampus were obtained using a microtome (Leica) and preserved at − 20 °C in an anti-freezing solution.

Cells and brain sections were fixed in 4% PFA for 10 min, permeabilized with 0.3% Triton X-100 for 10 min, and then incubated in 10% bovine serum albumin for 1 h at room temperature to block nonspecific binding. They were then incubated with primary antibodies at 4 °C overnight followed by incubation with secondary antibodies at room temperature for 1 h after washing with PBS. Sequentially, they were incubated with 4,6-diamidino-2-phenylindole (DAPI, Life Technologies, Mulgrave, VIC, Australia) for 10 min at room temperature. The primary antibodies used were Arginase-1 (Arg-1; 1:50; Santa Cruz, CA, USA), cluster of differentiation 206 (CD206; 1:200; Abcam, Cambridge, MA, USA), inducible nitric oxide synthase (iNOS; 1:100; Abcam), CD16/32 (1:200; BD Pharmingen, San Diego, CA, USA), ionized calcium-binding adapter molecule-1 (Iba-1; 1:200; WAKO, Osaka, Japan), mitogen-activated protein 2 (MAP2; 1:100; Millipore), NeuN (1:200; Millipore), microtubule-associated protein 1 light chain 3 (LC3; 1:200; Sigma-Aldrich), and lysosomal-associated membrane proteins 2 (LAMP2; 1:500; Millipore), CD206 (1:200; Abcam).

These cells or brain sections were imaged by confocal microscopy (Leica). Three to five regions of three brain sections were analyzed for each mouse, and five regions of each cell slide were analyzed. ImageJ software (NIH, Bethesda, MD, USA) was used for integrated optical density quantification.

### Western blot analysis

Mouse brains were obtained under deep anesthesia 1 and 3 days after surgery, and the ipsilateral hemisphere was used for Western blot analysis. For cells and brain tissue, the lysates were prepared using RIPA lysis buffer (Millipore) supplemented with a protease inhibitor cocktail (Roche, Basel, Switzerland). The Western blot protocol was performed as previously described [21]. The primary antibodies used were iNOS (1:500; Abcam), Arginase-1(Arg-1; 1:500; Santa Cruz), CD206 (1:800, Abcam), LC3B (1:1000, Sigma-Aldrich), LAMP2 (1:500; Millipore), phospho-mTOR (Ser 2448) (p-mTOR; 1:500; Cell Signaling Technology, Beverly, MA, USA), mTOR (1:1000, Cell Signaling Technology), FLAG (1:1000; Abcam), caspase3 (cas3; 1:500, Cell Signaling Technology), BCL2-associated X protein (bax; 1:1000; Abcam), B cell lymphoma 2 (bcl2;1:500; Cell Signaling Technology), GAPDH (1:1000; Santa Cruz), and β-actin (1:1000; Santa Cruz). The immunoblots were detected using an enhanced chemiluminescence kit (FD Technology, Shanghai, China) and obtained using an imaging system (Bio-Rad, Hercules, CA, USA) and then analyzed by ImageJ software.

### Real-time quantitative PCR

Mouse brains were obtained under deep anesthesia at 1 and 3 days after surgery, and the ipsilateral hemisphere was used. Total RNA from tissues and cells was extracted using TRIzol reagent (Invitrogen, Carlsbad, CA, USA) according to the manufacturer’s protocol. Single-strand cDNA was synthesized using a Zymoscropt First-strand cDNA Synthesis Kit (Zymo tool, Shanghai, China). Gene transcription was detected using a 7900HT sequence detection system (Applied Biosystems, Foster City, CA, USA) using specific primers according to the protocol of the SYBR Premix Ex Taq Kit (Takara, Dalian, China). The *GAPDH* gene was used as an endogenous control.

The primer sequences were as follows: TNF-α (FW, ACCCTCACACTCAGATCATCTT; RV, GGTTGTCTTTGAGATCCATGC), IL-1β (FW, CGCAGCAGCACATCAACAAGAGC; RV, TGTCCTCATCCTGGAAGGTCCACG), IL-6 (FW, GGTTGTCTTTGAGATCCATGC; RV, GGTCTTGGTCCTTAGCCACTC), TGF-β (FW, CACCGGAGAGCCCTGGATA; RV, TGTACAGCTGCCGCACACA), IL-10 (FW, GCGCTGTCATCGATTTCTCC; RV, TGGCCTTGTAGACACCTTGG), IGF-1 (FW, CTCTGCTTGCTCACCTTC; RV, CCTTCTCCTTTGCAGCTTC), and GAPDH (FW, AGGTCGGTGTGAACGGATTTG; RV, TGTAGACCATGTAGTTGAGGTCA).

### Statistical analysis

All results are presented as the mean ± SEM. Statistical comparisons were performed by unpaired Student’s *t* test or one-way analysis of variance (ANOVA) followed by Bonferroni post hoc test. *p <* 0.05 was considered statistically significant. Statistical analyses were carried out and charts were drawn using GraphPad Prism 5 (GraphPad Software, San Diego, CA, USA).

## Results

### Sestrin2 improved neurological functional recovery and reduced cell apoptosis after tMCAO in mice

To investigate the effects of sestrin2 treatment on the outcomes of cerebral ischemia and reperfusion, we used a tMCAO animal model. One microgram rh-sestrin2, 3 μg rh-sestrin2, or normal saline were administrated through intracerebroventricular injection instantly after tMCAO. Mice were tested and sacrificed 1 day and 3 days after tMCAO (Fig. 1a). To examine whether rh-sestrin2 was successfully injected and determine the time period for which rh-sestrin2 levels were maintained, we used Western blotting to semi-quantify FLAG-tagged rh-sestrin2 in the ipsilateral hemisphere at 1 and 3 days after tMCAO. Significant levels of FLAG-tagged rh-sestrin2 were detected, which were sustained 3 days after injection (Fig. 1d). We examined the neurological outcomes at 1 day and 3 days after tMCAO. The Clark and Longa neurological deficit scores showed that 1 μg sestrin2 attenuated neurobehavioral deficits at 1 day after tMCAO (*p <* 0.05) compared to that in the NS-treated group while 3 μg sestrin2 did not (Fig. 1b, c). The neurological deficits showed no significant difference between the NS- and sestrin2-treated groups at 3 days after tMCAO (Fig. S1). In addition, 1 μg sestrin2 also ameliorated infarct percentage in the ischemic hemisphere at 1 day after tMCAO compared to the NS group (*p <* 0.05), while 3 μg sestrin2 increased the infarction percentage compared to the low-dose group (Fig. 1e, f). Therefore, we selected 1 μg of sestrin2 to conduct the following experiments in order to explore the underlying protective mechanisms.

**Fig. 2:**
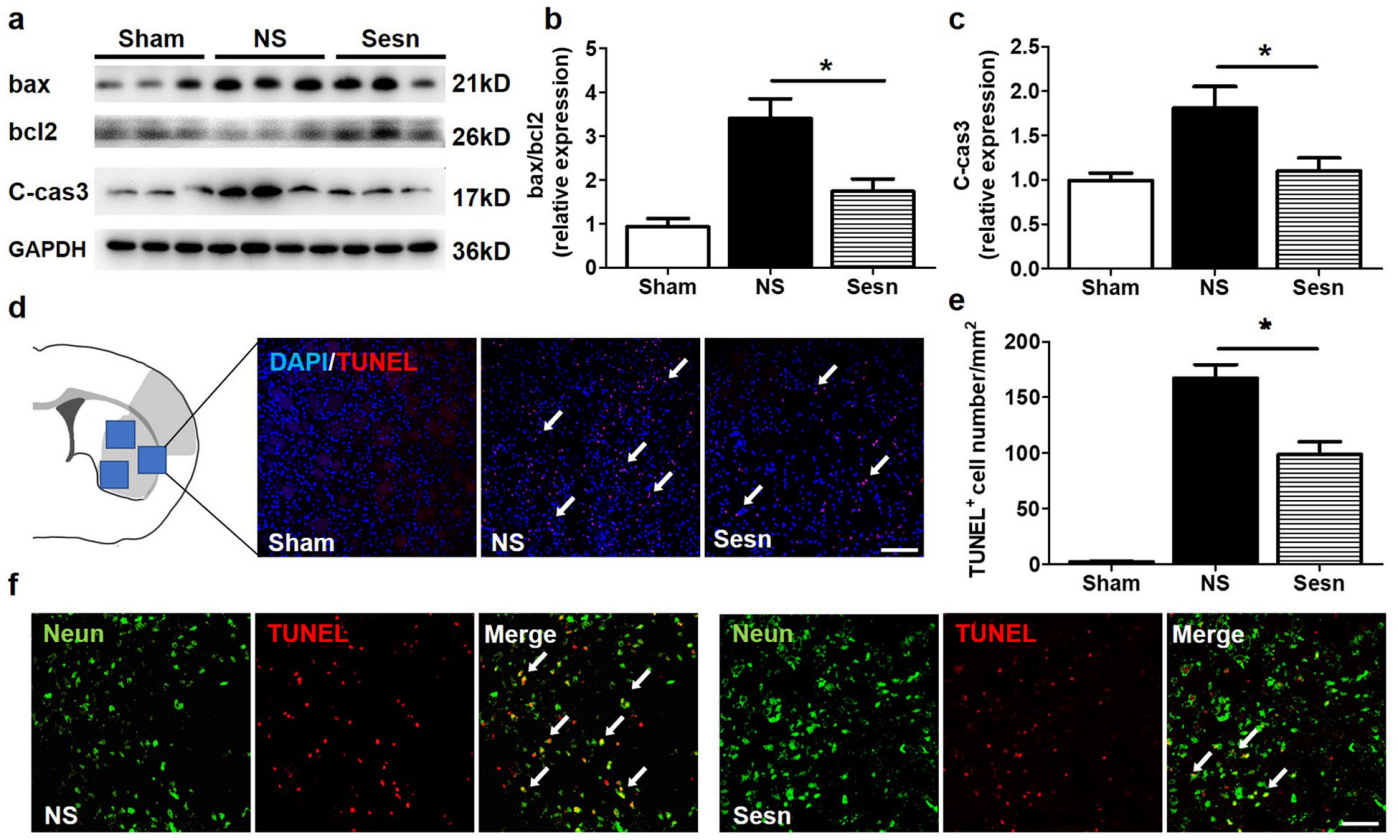
Sestrin2 reduced cell apoptosis after tMCAO. **a**–**c** Western blot and quantification analysis show the expression of bax, bcl2, and cleaved caspase3 in Sham, NS, and Sesn group at 3 days after tMCAO. C-cas3 = cleaved caspase3. *N* = 4–5 per group. Data are presented as mean ± SEM, *p < 0.05, NS vs. Sesn. **d**, **e** TUNEL staining and bar graph show the number of TUNEL^+^ (red) cells in NS and Sesn groups 1 day after tMCAO. White arrows point at TUNEL^+^/DAPI^+^ cells. Scale bar = 100 μm. *N* = 5 per group. Data are presented as mean ± SEM, *p < 0.05, NS vs. Sesn. **f** Representative photos of TUNEL^+^/Neun^+^ cells in NS and Sesn groups. White arrows point at TUNEL^+^/Neun^+^ cells. Scale bar = 50 μm

To examine whether sestrin2 prevents neuronal apoptosis, we analyzed bcl-2, bax, and cleaved caspase-3 protein levels by Western blotting and found that sestrin2 decreased bax/bcl-2 and cleaved caspase-3 protein levels compared to the NS group (*p* < 0.05, Fig. 2a–c). TUNEL and TUNEL/NeuN double immunostaining showed that sestrin2 decreased the number of apoptotic cells, mainly neurons, in the peri-infarct region (Fig. 2d–f). These results suggested that sestrin2 could improve neurological functional recovery by preventing cell apoptosis after tMCAO.

### Sestrin2 reduced microglial activation and promoted M2 phenotype transition after tMCAO

**Fig. 3:**
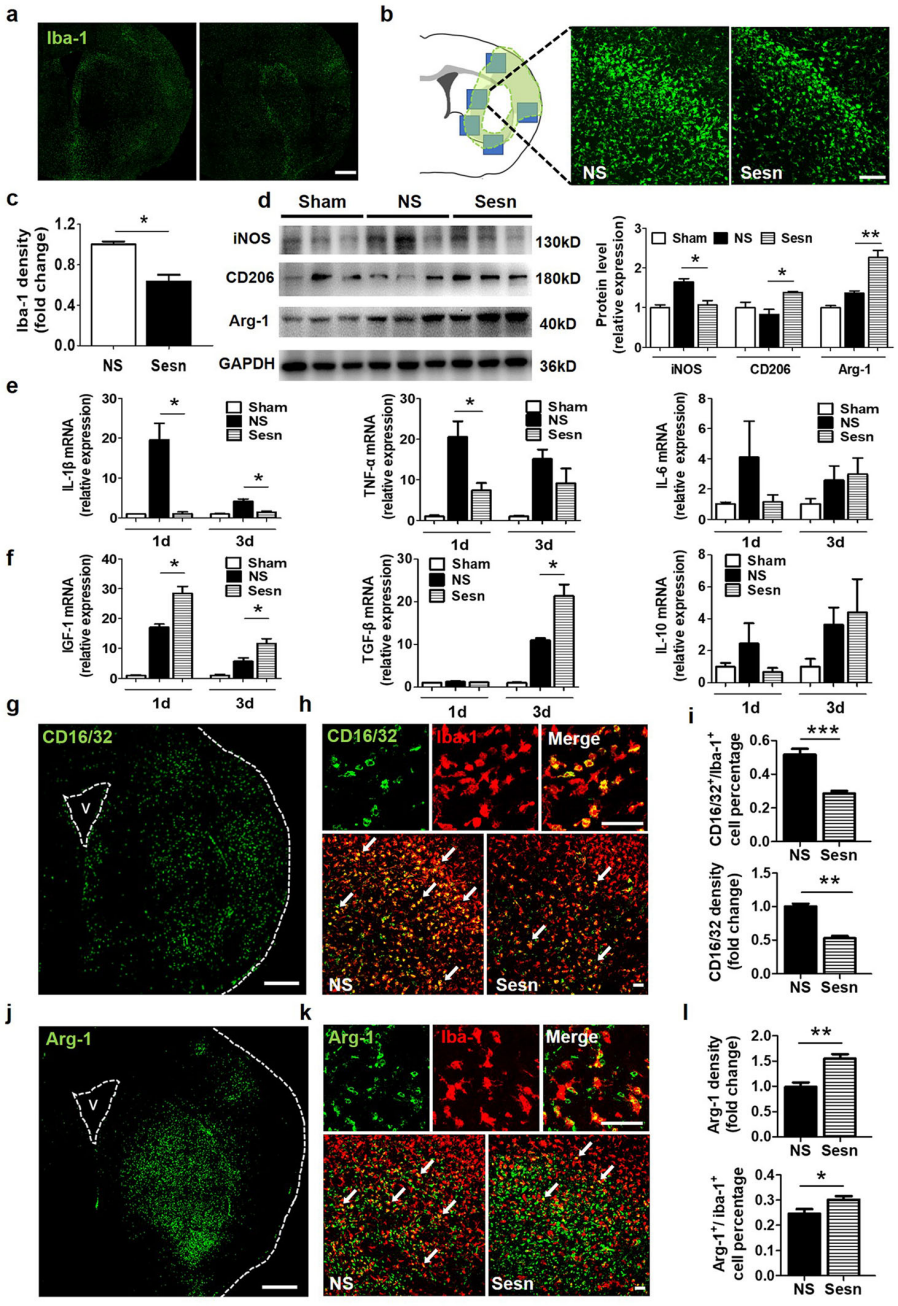
Sestrin2 reduced microglia activation and promoted M2 phenotype polarization after tMCAO. **a** Representative images of Iba-1 in the ipsilateral hemisphere of NS and Sesn groups. Scale bar = 200 μm. **b** Schematic diagram illustrating the peri-infarct area (green) and the photographed region (boxed areas) of immunostaining images for Iba-1. Scale bar = 100 μm. **c** Quantification of Iba-1 relative density in NS and Sesn groups. *N* = 4 per group. **d** Western blot and quantification analysis show the expression of iNOS, CD206, and Arginase-1 in Sham, NS, and Sesn group at 3 days after tMCAO. *N* = 4–5 per group. Arg-1 = Arginase-1. **e**, **f** The results of the real-time PCR show mRNA levels of IL-1β, IL-6, TNF-α, IGF-1, IL-10, and TGF-β in Sham, NS, and Sesn groups at 1 day and 3 days after tMCAO. *N* = 4–6 per group. **g** Immunostaining images of CD16/32 in the ipsilateral hemisphere, white dotted lines show borderlines of ipsilateral hemisphere. V = ventricular. Scale bar = 400 μm. **h** Immunostaining images of CD16/32 (green) and Iba-1 (red) in NS and Sesn group. White arrows point at CD16/32^+^/Iba-1^+^ cells. Scale bar = 200 μm. **i** Quantification of CD16/32 fluorescent intensity and CD16/32^+^/Iba-1^+^ cell number in NS and Sesn groups. *N* = 4 per group. **j** Immunostaining images of Arginase-1 in ipsilateral hemisphere, white dotted lines show borderlines of ipsilateral hemisphere. V = ventricular. Arg-1 = Arginase 1. Scale bar = 400 μm. **k** Immunostaining of Arginase-1 (green) and Iba-1 (red) in NS and Sesn group. White arrows point at Arginase-1^+^/Iba-1^+^ cells. Scale bar = 50 μm. **l** Quantification of Arginase-1 fluorescent intensity and Arginase-1^+^/Iba-1^+^ cell number in NS and Sesn groups. Arg-1 = Arginase-1. Data are presented as mean ± SEM, *p < 0.05, **p < 0.01, ***p < 0.001

It has been shown that sestrin2 reduces inflammation by regulating anti-inflammatory and pro-inflammatory mediator release [17]. Since microglia are the canonical inflammatory cells in the brain, we quantified the number of microglia by Iba-1 immunofluorescent staining. The results showed that activated microglia were mainly located at the border of the ischemic lesion, and sestrin2 significantly reduced microglia activation compared to the NS group (Fig. 3a–c).

Microglia exhibit dynamic polarization over time and can switch between the M1 or M2 phenotypes according to environmental stimuli [25]. M1/M2 microglia/macrophages are distinguished by the expression of feature genes. Western blot results showed that the levels of the M1-related marker iNOS decreased and those of the M2 related markers Arginase-1 and CD206 increased after sestrin2 treatment (Fig. 3d, e). The M1 microglia-related marker CD16/32 was highly expressed in Iba-1 positive cells (microglia/macrophage) around the ischemic lesion. Sestrin2 significantly reduced CD16/32 expression and increased the expression of the M2 related marker Arginase-1 in microglia 3 days after tMCAO (Fig. 3g–l).

We also measured the M1-related pro-inflammatory and M2-related anti-inflammatory factors at the transcriptional level by quantitative real-time PCR at 1 and 3 days after tMCAO. Results showed that sestrin2 decreased the mRNA levels of the pro-inflammatory factor IL-1β at 1 and 3 days, and TNF-α expression at 1 day after tMCAO compared to that in the NS group (Fig. 3e). IL-6 expression did not change after sestrin2 treatment. Moreover, sestrin2 significantly increased the mRNA levels of anti-inflammatory mediators IGF-1 at 1 and 3 days, and TGF-β at 3 days after tMCAO. However, sestrin2 had the potential to decrease IL-10 expression at 1 day after tMCAO (Fig. 3f). These results suggest that sestrin2 could affect microglial phenotype transition to promote neurological functional recovery after tMCAO.

### Sestrin2 promoted the transition of microglia from M1 to M2 phenotype after OGD to exert neuroprotective function in vitro

**Fig. 4:**
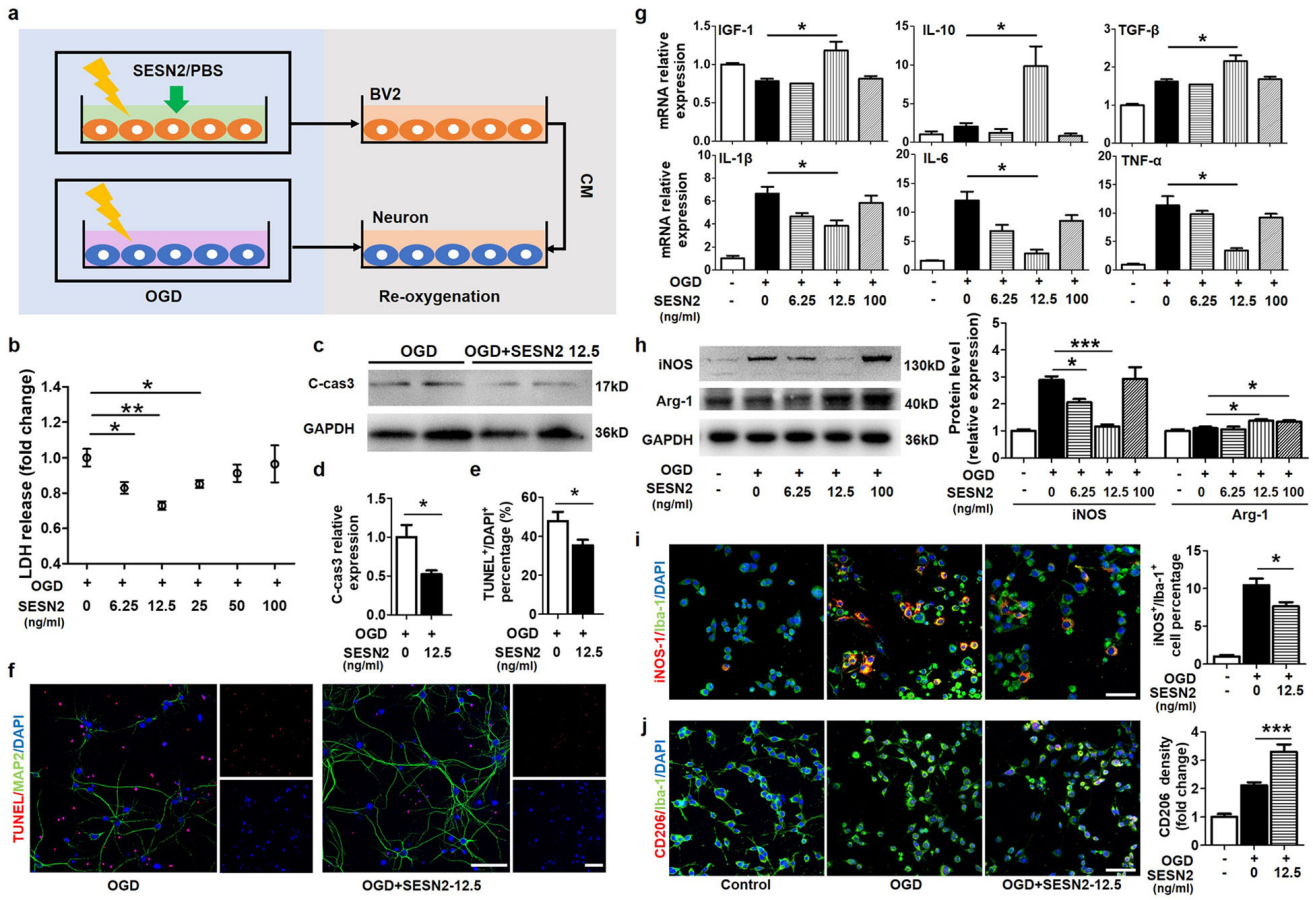
Sestrin2 promotes microglial polarization to M2 phenotype to exert neuroprotection after oxygen and glucose deprivation and re-oxygenation*.* **a** Diagram of the in vitro experimental design. *SESN2* Sestrin2, *PBS* phosphate buffer saline, *CM* condition medium. **b** LDH assay showing death of neurons treated with conditioned medium from OGD-treated BV2 cells supplemented with different doses of sestrin2. **c**, **d** Western blot and quantification for cleaved caspase3 in neurons of two groups treated with conditioned medium from BV2 cells culture under OGD conditions or with conditioned medium from OGD-treated cells supplemented with 12.5 ng/ml sestrin2 (OGD + SESN 12.5). C-*cas3* cleaved caspase3. **e**, **f** Immunostaining images of TUNEL (red), MAP2 (green), and DAPI (blue) in OGD and OGD + SESN 12.5 groups. Scale bar = 50 μm. The bar graph shows the percentage of TUNEL^+^ cells/ DAPI^+^ cells. **g** The results of the real-time PCR show the mRNA levels of the pro-inflammatory mediators IL-1β, IL-6, and TNF-α and anti-inflammatory mediators IGF-1, IL-10, and TGF-β in BV2 cells treated after OGD with 6.25–100 ng/ml sestrin2 and NS. **h** Western blot and quantification analysis show the expression of M1-related marker (iNOS) and M1-related marker (Arginase-1) in BV2 cells treated after OGD with 6.25–100 ng/ml sestrin2 and NS. **i** Immunostaining images of M1 related marker iNOS (red), Iba-1 (green), and DAPI (blue) in control, OGD and OGD + SESN 12.5 groups. Scale bar = 50 μm. Quantification of iNOS^+^ cell number and its percentage to Iba-1^+^ cell number in OGD and OGD+SESN 12.5 groups. **j** Immunostaining for the M2-related marker CD206 (red), Iba-1 (green) and DAPI (blue) of cells in control, OGD, and OGD + SESN 12.5 groups. Scale bar = 50 μm. Quantification of CD206^+^ cell number and its relative density in OGD and OGD + SESN 12.5 group. *N* = 4–6 per group. Data are presented as mean ± SEM, *p < 0.05, **p < 0.01, ***p < 0.001

To investigate the neuroprotective effects of sestrin2 in vitro, we performed OGD of primary neurons for 45 min, and then, we treated primary neurons with re-oxygenated condition medium of BV2 cells after administration of different doses of recombined sestrin2 (6.25–100 ng/ml) or PBS as control, during OGD (Fig. 4a). The condition medium of BV2 cells treated with a dose of 6.25–25 ng/ml sestrin2 significantly increased neuronal survival as determined by the LDH assay; 50–100 ng/ml sestrin2 lost the protective effect but did not worsen the injury (Fig. 4b). Furthermore, the levels of cleaved caspase-3 decreased in the OGD + SESN12.5 group compared to those in the control group (Fig. 4c, d). MAP2 and TUNEL double immunostaining showed that the condition medium of OGD BV2 cells treated with 12.5 ng/ml sestrin2 significantly decreased the percentage of TUNEL-positive cells (Fig. 4e, f).

We then investigated the effects of sestrin2 on microglial phenotype transition, and BV2 cells were collected after OGD for further analysis. We measured the mRNA levels of pro-inflammatory factors by quantitative real-time PCR in different groups. The results showed that IL-1β, IL-6, and TNF-α mRNA levels decreased in a dose-dependent manner, and 12.5 ng/ml sestrin2 significantly reduced their levels (*p* < 0.05). We also found that 12.5 ng/ml sestrin2 upregulated IGF-1, IL-10, and TGF-β mRNA expression (*p* < 0. 05), while 6.25 ng/ml and 100 ng/ml had no such effect (Fig. 4g). We also performed immunostaining for the M1-related marker iNOS and the M2-related marker CD206. The results demonstrated that 12.5 ng/ml sestrin2 reduced the number of iNOS^+^ cells and the percentage of iNOS^+^/Iba-1^+^ cells, and increased CD206 immunofluorescent density (Fig. 4i, j). Meanwhile, the protein levels of iNOS decreased and those of Arginase-1 increased in the group treated with 12.5 ng/ml sestrin2 compared to those in the OGD group (*p* < 0.05). Interestingly, 100 ng/ml sestrin2 treatment also upregulated iNOS protein expression (*p <* 0.05, Fig. 4h). Considering these, our results indicated that sestrin2 could indirectly reduce neuronal damage by shifting microglial polarization from the M1 to the M2 phenotype in a dose-dependent manner.

### Sestrin2 induced M2 phenotype transition by inhibiting the mTOR signaling pathway and restoring autophagic flux in BV2 cells

**Fig. 5:**
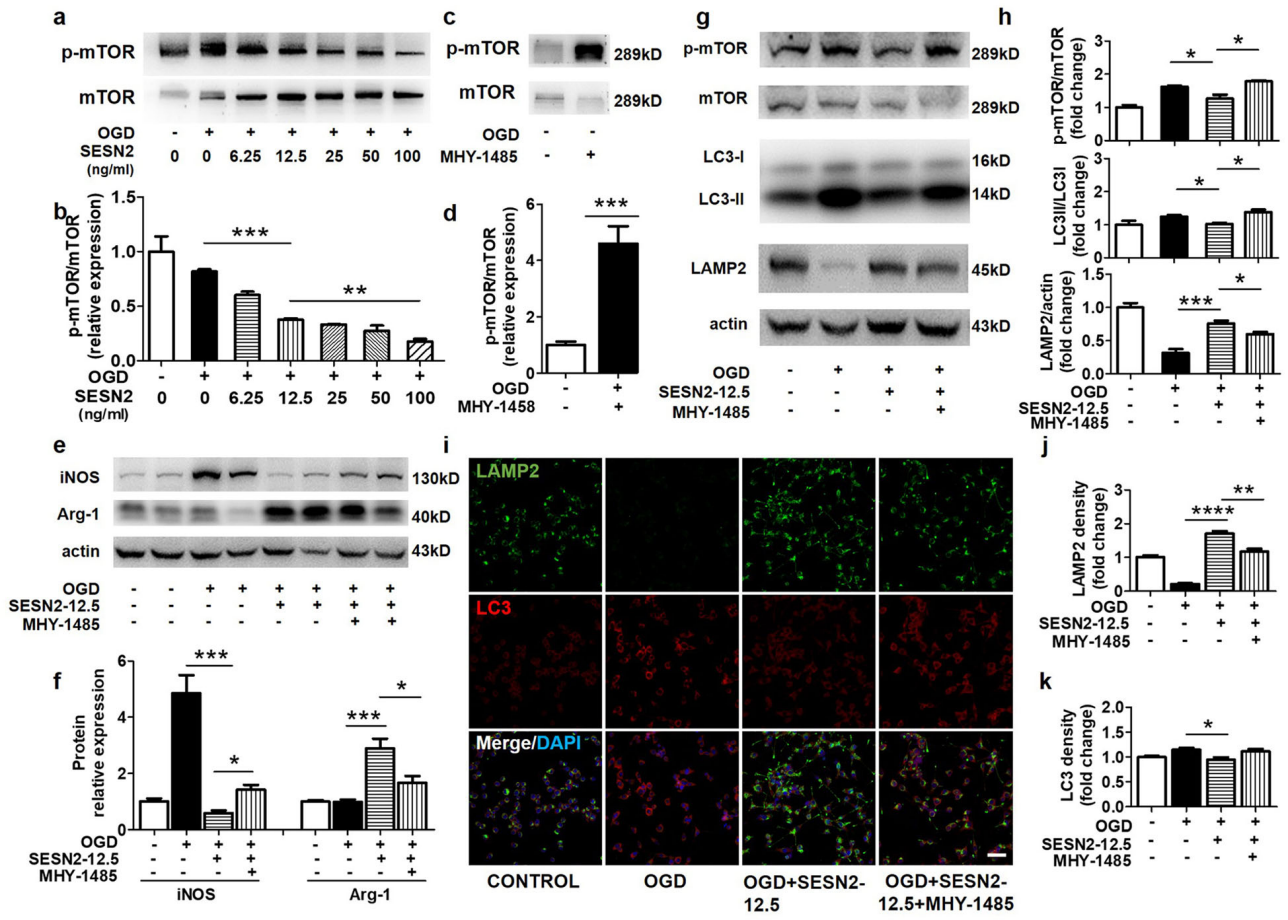
Sestrin2 induced microglial M2 polarization by inhibiting mTOR signaling and restoring autophagic flux. **a**, **b** Western blot and the quantification for p-mTOR and mTOR in OGD/R treated BV2 cells supplemented with different doses of sestrin2. **c**, **d** Western blot and the quantification for p-mTOR and mTOR in OGD treated BV2 cells supplemented with or without MHY-1485. **e**, **f** Western blot and the quantification for iNOS and Arginase-1 in OGD treated BV2 cells supplemented with different doses of sestrin2 and MHY-1485. *Arg*-*1* Arginase-1. **g**, **h** Western blot and the quantification for p-mTOR/mTOR, LC3II/LC3I, LAMP2/actin in control, OGD, OGD + SESN2, and OGD + SESN2 + MHY-1485 groups. **i** Immunostaining images for LAMP2 (green) and LC3 (red) of BV2 cells in control, OGD, OGD + SESN2, and OGD + SESN2 + MHY-1485 groups. Scale bar = 25 μm. **j**, **k** Quantification of LAMP2 and LC3 relative density in control, OGD, OGD + SESN2, and OGD + SESN2 + MHY-1485 groups. *N* = 4–6 per group. Data are presented as mean ± SEM, *p < 0.05, **p < 0.01, ***p < 0.001

Previous studies have shown that sestrin2 inhibited the mTOR complex 1 (mTORC1) signaling pathway, and mTORC1-mediated macrophage phenotype transition and function [19]. To further confirm the role of mTOR signaling in BV2 cells treated with sestrin2, we treated BV2 cells with an mTOR activator MHY-1485 at the beginning of OGD. First, we found that sestrin2 suppressed mTOR phosphorylation in a dose-dependent manner in BV2 cells after OGD (Fig. 5a, b), which was consistent with previous studies [26]. Second, we found that MHY-1485 treatment increased mTOR phosphorylation in BV2 cells (Fig. 5c, d). MHY-1485 treatment increased iNOS expression but reduced Arginase-1 expression, which suggested that mTOR activation counteracted the effect of sestrin2 on the ratio of M1/M2 phenotypes (Fig. 5e, f).

**Fig. 6:**
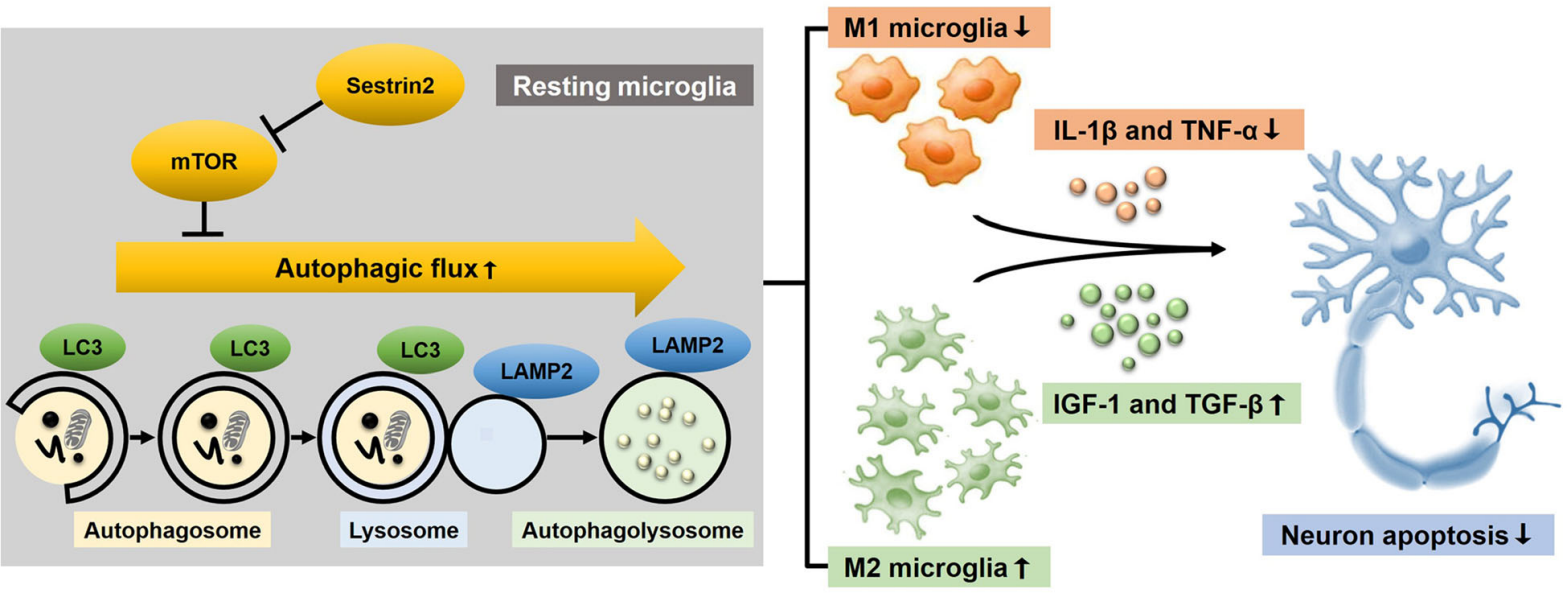
Schematic of the neuroprotective role of sestrin2. After ischemia/hypoxia, sestrin2 promoted microglial polarization to M2 phenotype by restoring autophagic flux through mTOR pathway inhibition to prevent neuronal apoptosis

In addition, mTOR is a major repressor of autophagy [27]. Previous studies have shown that there is a relationship between autophagy and microglia polarization, and impaired autophagy promotes M1 polarization of macrophages [28, 29]. In order to investigate whether sestrin2 affects the autophagic pathway through mTOR, we examined the expression of the autophagy-related proteins LAMP2 and LC3. Western blot results showed that the levels of LAMP2 decreased and the LC3II/I ratio increased in the OGD group compared to those in the control group (Fig. 5g, h). Consistent with the Western blot results, double immunostaining for LC3 and LAMP2 showed that sestrin2 decreased the levels of LC3 but increased LAMP2 expression compared to the OGD group, which could be reversed by the mTORC1 activator MHY-1485 (Fig. 5i–k). These results suggest that the autophagy is activated, and the autophagosome degradation pathway is inhibited by OGD, which indicates that autophagic flux is impaired. Sestrin2 administration restored autophagic flux by inhibiting over-activated autophagy and enhancing autophagosome degradation through lysosomes. This effect was partially blocked by mTOR activation. Taken together, these results indicate that sestrin2 inhibits mTOR to restore autophagic flux, which participates in the transition of microglia polarization to the M2 phenotype (Fig. 6).

## Discussion

In the present study, we demonstrated that sestrin2 regulates microglial polarization switch from the M1 to M2 phenotype via mTOR suppression and autophagic flux modulation, thus dampening the inflammatory response during the acute phase of cerebral ischemic injury. We verified the neuroprotective role of sestrin2 and found it to be dose-dependent in vivo. Exogenous administration of rh-sestrin2 (1 μg) exerted neuroprotective effects up to 3 days after tMCAO. We also demonstrated that 1 μg rh-sestrin2 treatment significantly reduced apoptosis.

Stroke-induced microglial activation plays either neurotoxic/pro-inflammatory or neuroprotective/anti-inflammatory roles depending on the balance between M1 and M2 microglia. Increasing evidence indicates that microglia are a highly heterogeneous cell population [30]. Novel technologies, including RNA-seq analysis, have revealed that microglial transcriptomic and proteomic profiles could be drastically different based on criteria such as the region, time, disease, and state [31]. Simple classification as M1 or M2 phenotype does not accurately describe the heterogeneity of microglia; however, this classification is still accepted and used, especially in the clinical treatment of stroke and neurodegenerative disease [32, 33]. Therefore, this simplified classification method is still instructive. Sestrin2 has an anti-inflammatory role in macrophages during sepsis, hepatitis, colitis, and myocardial infarction [17]. In this study, we found that sestrin2 reduced the expression of pro-inflammatory factors IL-1β and TNF-α, while simultaneously enhanced the expression of anti-inflammatory factors TGF-β and IGF-1 in microglia. These results were consistent with less activated microglia and a phenotype shift from pro-inflammatory M1 microglia to the anti-inflammatory M2 phenotype at 3 days post-stroke. We chose this assessment time point because temporal analyses of post-ischemic microglial phenotypes revealed that M2 microglia increased at 1 to 3 days temporally and then, gradually decreased in the following days, while M1 microglia gradually increased in the first 14 days after ischemic stroke [6, 11, 34]. Other studies have also reported that inflammatory brain injuries are critical in condition till 3 days after stroke [25, 35]. We found M1-specific marker CD16/32-positive cells with amoeboid morphology were mainly localized to the surrounding ischemic border area, while Arginase-1-positive M2 microglia with thick ramifications mainly located in the ischemic core at 3 days after tMCAO. Our results showed that sestrin2 treatment promoted the M2 phenotype but reduced the M1 phenotype to exert anti-inflammatory function. Furthermore, sestrin2 treatment did not affect the mRNA levels of the inflammatory mediators IL-6 and IL-10. This could be explained by the fact that IL-6 deficient mice demonstrated no difference in infarct size and neurological function after transient CNS ischemia, which suggests that IL-6 does not have a direct influence [36]. The M2 cells could be further divided into M2a, M2b, and M2c subtypes. M2b cells are marked by high expression of IL-10 [37], indicating that sestrin2 may promote some specific subtypes.

Immunostaining showed that some Arginase-1-positive cells in the ischemia core did not co-localize with Iba-1. M1 and M2 signature markers are shared between microglia and macrophages. Arginase-1 is not only expressed by resident microglia, but also by infiltrating macrophages post-stroke [6], suggesting that these cells might be infiltrating M2 macrophages. It has been reported that activated microglia and recruited macrophages from circulation were not antigenically distinguishable [38]. To exclude the effects of macrophage infiltration, we used the microglial cell line BV2 for further analysis. Sestrin2 promoted BV2 cell polarization from the pro-inflammatory M1 phenotype toward the anti-inflammatory M2 phenotype, which has a neuroprotective role.

mTOR plays a central role in cell metabolism, growth, survival, and degeneration [39]. Increasing evidence has shown that sestrin2 can protect cells by reducing oxidative stress and apoptosis in diabetes, cancer, and neurodegeneration, most of which target mTOR as a downstream factor [26, 40]. Sestrin2 can suppress mTOR signaling by activating AMPK and tuberous sclerosis protein complex 2 (TSC2) phosphorylation during stress [41, 42]. Our results suggest that sestrin2 suppresses mTOR phosphorylation. mTOR signaling is also involved in the regulation of immune reactions in many neurologic diseases [43]. Suppressing mTOR signaling inhibits neuronal inflammation in traumatic brain injury and Parkinson’s disease [44, 45]. In cultured macrophages, mTORC1 signaling inhibited mitophagy, leading to the accumulation of dysfunctional and pro-apoptotic mitochondria and promoted cell death. Inhibition of mTOR signaling has been shown to reduce the deleterious activation of macrophages and promoted anti-inflammatory M2 polarization in macrophages [46]. It has been shown that blockade of mTORC1 signaling enabled microglia to polarize toward the M2 phenotype to exert anti-inflammatory effects after stroke [47, 48]. Our study indicated that sestrin2 suppressed mTOR signaling to increase the M2/M1 ratio and exhibit anti-inflammatory function.

mTOR has also been known as a key regulator of autophagy and therefore, of cellular homeostasis. Autophagy is a lysosomal-dependent catabolic process by which cellular misfolded proteins and impaired organelles are enclosed into double-membrane autophagosomes, which are then fused with lysosomes for degradation and recycling [49, 50]. Autophagic flux dysfunction is characterized by the retention of damaged components and overburden autophagosomes, leading to cellular stresses and inflammation [51, 52]. Impaired autophagy promotes pro-inflammatory M1 macrophage polarization and increases immune response in obese mice [29]. Autophagic flux in tumor-associated macrophages could promote M2 polarization [53]. It has been shown that TNF-α-inhibited autophagy drives microglial polarization toward the M1 phenotype by activating the Akt/mTOR pathway, and autophagy activation enhances M2 polarization to attenuate neurotoxicity [28]. Our results indicated that dysregulated autophagic flux after cerebral ischemic injury was restored by sestrin2 treatment. Furthermore, the mTOR activator MHY-1485 partially blocked this effect, suggesting that sestrin2 may modulate the autophagy-lysosome pathway through mTOR inhibition in microglia.

It has been noted that OGD increased the levels of LAMP2 in endothelial cells [54], but decreased LAMP2 expression in neurons [55]. Our results supported that OGD decreased LAMP2 expression in BV2 cells, indicating a defect in lysosome function. We found that mTOR activation by MHY-1485 administration upregulated the LC3II/I ratio, which may be due to the complex mTOR signaling regulation of autophagic flux by to. mTOR regulates autophagy not only through direct phosphorylation of unc-51 like autophagy activating kinase 1 (Ulk1) during autophagy initiation but also through termination of autophagy and reformation of lysosomes [56, 57]. The mTORC1 activator MHY-1485 has been shown to have an inhibitory effect on the autophagic process by inhibiting fusion of autophagosomes and lysosomes, thus leading to the accumulation of LC3II protein and enlarged autophagosomes [58], which suggests that autophagosome accumulation is not only due to the induction of autophagy but also due to impairment of autophagic flux. Altogether, the in vitro study demonstrated that sestrin2 promoted microglia shifting from M1 to M2 phenotype, which indirectly led to reduced neuronal apoptosis by inhibiting mTOR signaling and restoring autophagic flux. However, the mTOR activator MHY-1485 only partially counteracted the function of sestrin2, indicating that other mechanisms may be involved. The determination of the human sestrin2 crystal structure allows the identification of three functional domains [59]. At the C-terminal, there is a GATOR2-interacting surface that allows for subsequent inhibition of mTORC1 and impairment of autophagic flux. In addition, Sesn-A at the N-terminal reduces the levels of ROS through its helix-turn-helix oxidoreductase motif. This anti-oxidative effect of sestrin2 might also reduce iNOS expression and regulate autophagy and inflammation [60].

Our in vivo study showed that a higher dose of rh-sestrin2 (3 μg) did not reduce neurological deficits, and even increased the infarct area compared to the lower dose (1 μg). At the same time, the in vitro study showed that 100 ng/ml sestrin2 upregulated M1 and M2 marker expression concurrently, and that BV2-CM did not protect against neuronal injury. Our results indicated that only a moderate dose of exogenous sestrin2 coordinated the balance between M1 and M2 phenotypes to support a neuroprotective role. Moderate autophagy exerts protective effects against various pathologies, while excessive or defective autophagy leads to cell death [61]. Mild inflammation is also good for cell metabolism, while excessive inflammation leads to injury [62]. This dose-dependent function of sestrin2 could be explained by the complex regulation of inflammation and autophagic flux. We hypothesized that the “overdose” of sestrin2 could further inhibit mTOR signaling leading to suppressive autophagy, both its induction and degradation, and eventually induce microglial cell death in the meantime of M2 phenotype change. Further studies are needed to elucidate this “overdose” function and reveal more about the relationship between autophagic flux and microglia polarization.

## Conclusions

Our current study revealed that sestrin2 promoted microglia shift from the M1 to the M2 phenotype for neuroprotection after ischemic brain injury by suppressing mTOR signaling and restoring autophagic flux in a dose-dependent manner, indicating a novel immunomodulatory approach for ischemic stroke therapy.

## Supplementary information

**Additional file 1: Fig. S1.** a and b Bar graphs showing the results of the Clark and Longa neurological deficit scores at 3 days after tMCAO in Sham, NS, Sesn, H-sesn groups. *N* = 10 per group. Data are presented as mean ± SEM.

**Additional file 2.** English editing certificate.

## Data Availability

The datasets obtained and/or analyzed during the current study as well as any additional data are available from the corresponding author upon reasonable request.

## Abbreviations

AMPK: Adenosine 5′-monophosphate-activated protein kinase
Arg-1: Arginase-1
bax: BCL2-associated X protein
bcl2: B cell lymphoma 2
BV2-CM: BV2 re-oxygenation condition medium
C-cas3: Cleaved caspase 3
DAPI: 4′, 6-Diamidino-2-phenylindole
CD16/32: Cluster of differentiation 16/32
CD206: Cluster of differentiation 206
DMEM: Dulbecco’s modified Eagle’s medium
HO-1: Heme oxygenase-1
IL: Interleukin
Iba-1: Ionized calcium-binding adaptor molecule-1
iNOS: Inducible nitric oxide synthase
IGF-1: Insulin growth factor-1
LAMP2: Lysosomal-associated membrane proteins 2
LC3: Microtubule-associated protein 1 light chain 3
LDH: Lactate dehydrogenase
LKB1: Liver kinase B1
MAP 2: Mitogen-activated protein 2
MAPK: Mitogen-activated protein kinase
mTOR: Mammalian target of rapamycin
mTORC1: mTOR complex 1
Nrf2: NF-E2-related factor 2
NS: Normal saline
OGD: Oxygen-glucose deprivation
PBS: Phosphate-buffered saline
TGF-β: Transforming growth factor β
TSC2: Tuberous sclerosis protein complex 2
tMCAO: Transient middle cerebral artery occlusion
TNF-α: Tumor necrosis factor α
Ulk1: Unc-51-like autophagy activating kinase 1
