# Supplementary figures and images for: Sestrin2 regulates microglia polarization through mTOR-mediated autophagic flux to attenuate inflammation during experimental brain ischemia
Source: J Neuroinflammation. 2020 Nov 5;17:329. doi: 10.1186/s12974-020-01987-y (PMC7643276; doi:10.1186/s12974-020-01987-y)

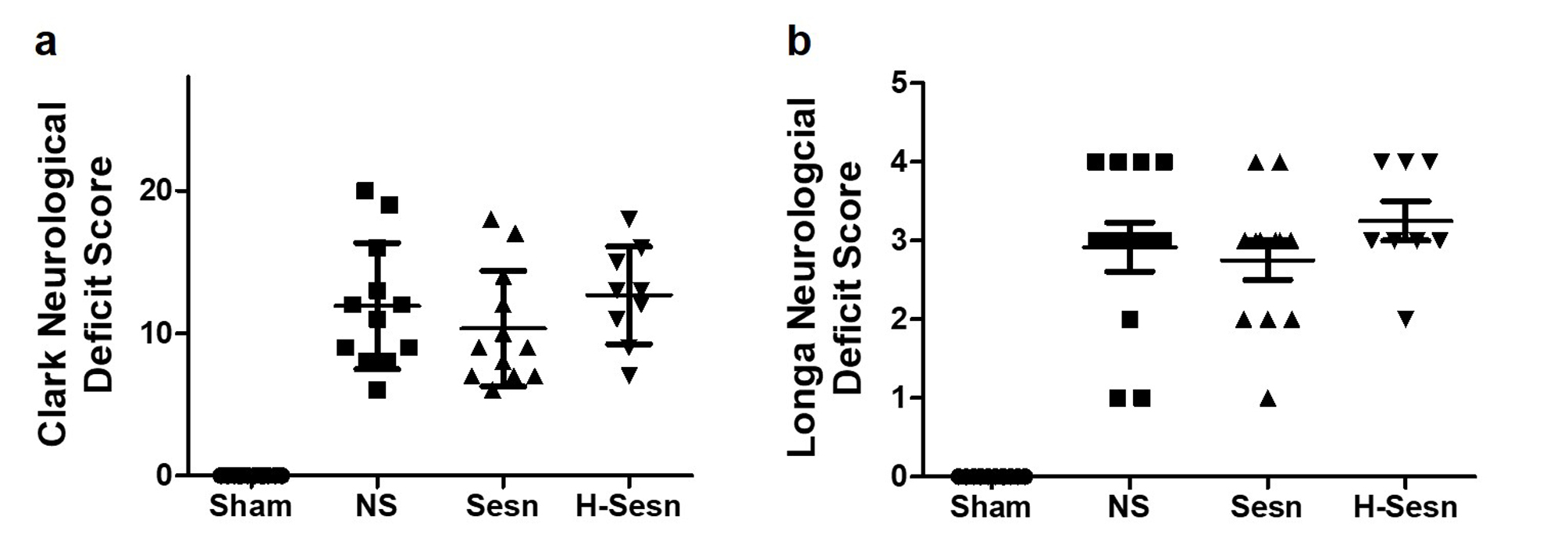

Supplement: Supplementary file 1 — Additional file 1: Fig. S1. a and b Bar graphs showing the results of the Clark and Longa neurological deficit scores at 3 days after tMCAO in Sham, NS, Sesn, H-sesn groups. N = 10 per group. Data are presented as mean ± SEM. [file 12974_2020_1987_MOESM1_ESM.jpg]
