# Supplementary material for: Sestrin2 regulates microglia polarization through mTOR-mediated autophagic flux to attenuate inflammation during experimental brain ischemia
Source: J Neuroinflammation. 2020 Nov 5;17:329. doi: 10.1186/s12974-020-01987-y (PMC7643276; doi:10.1186/s12974-020-01987-y)
Supplement: Supplementary file 2 — Additional file 2. English editing certificate. [file 12974_2020_1987_MOESM2_ESM.pdf]

Wiley Editing Services

# ENGLISH EDITING CERTIFICATE

This document certifies that the manuscript listed below was edited for proper English language, grammar, punctuation, spelling, and overall style by one or more of the highly qualified native English speaking editors at Wiley Editing Services

## Manuscript title

Sestrin2 regulates microglia polarization through mTOR mediated autophagic flux to attenuate inflammation during experimental brain ischemia

## Authors

Tingting He, MD; Wanlu Li, PhD; Yaying Song, MD; Zongwei Li, MS, Yaohui Tang, PhD; Zhijun Zhang, PhD; and Guo-Yuan Yang

## Order No

SPHUH_3

## Date Issued

September 02, 2020

This document certifies that the manuscript listed above was edited for proper English language, grammar, punctuation, spelling, and overall style. Neither the research content nor the authors' intentions were altered in any way during the editing process. Documents receiving this certification should be English-ready for publication; however, the author has the ability to accept or reject our suggestions and changes. If you have any questions or concerns about this document or certification, please contact help@wileyeditingservices.com.

Wiley Publishing Services is a service of Wiley Publishing. Wiley's Scientific, Technical, Medical, and Scholarly (STMS) business serves the world's research and scholarly communities, and is the largest publisher for professional and scholarly societies. Wiley is committed to providing high quality services for researchers. To find out more about Wiley Editing Services, visit http://wileyeditingservices.com. To learn more about our other author services provided by Wiley Publishing, visit https://authorservices.wiley.com/

WILEY
